# A novel film–pore–surface diffusion model to explain the enhanced enzyme adsorption of corn stover pretreated by ultrafine grinding

**DOI:** 10.1186/s13068-016-0602-2

**Published:** 2016-08-30

**Authors:** Haiyan Zhang, Longjian Chen, Minsheng Lu, Junbao Li, Lujia Han

**Affiliations:** College of Engineering, China Agricultural University (East Campus), P.O. Box 191, 17 Qing-Hua-Dong-Lu, Hai-Dian District, Beijing, 100083 People’s Republic of China

**Keywords:** Enzyme adsorption, Film–pore–surface diffusion, Kinetic model, Ultrafine grinding

## Abstract

**Background:**

Ultrafine grinding is an environmentally friendly pretreatment that can alter the degree of polymerization, the porosity and the specific surface area of lignocellulosic biomass and can, thus, enhance cellulose hydrolysis. Enzyme adsorption onto the substrate is a prerequisite for the enzymatic hydrolysis process. Therefore, it is necessary to investigate the enzyme adsorption properties of corn stover pretreated by ultrafine grinding.

**Results:**

The ultrafine grinding pretreatment was executed on corn stover. The results showed that ultrafine grinding pretreatment can significantly decrease particle size [from 218.50 μm of sieve-based grinding corn stover (SGCS) to 17.45 μm of ultrafine grinding corn stover (UGCS)] and increase the specific surface area (SSA), pore volume (PV) and surface composition (SSA: from 1.71 m^2^/g of SGCS to 2.63 m^2^/g of UGCS, PV: from 0.009 cm^3^/g of SGCS to 0.024 m^3^/g of UGCS, cellulose surface area: from 168.69 m^2^/g of SGCS to 290.76 m^2^/g of UGCS, lignin surface area: from 91.46 m^2^/g of SGCS to 106.70 m^2^/g of UGCS). The structure and surface composition changes induced by ultrafine grinding increase the enzyme adsorption capacity from 2.83 mg/g substrate of SGCS to 5.61 mg/g substrate of UGCS. A film–pore–surface diffusion model was developed to simultaneously predict the enzyme adsorption kinetics of both the SGCS and UGCS. Satisfactory predictions could be made with the model based on high *R*^2^ and low *RMSE* values (*R*^2^ = 0.95 and *RMSE* = 0.16 mg/g for the UGCS, *R*^2^ = 0.93 and *RMSE* = 0.09 mg/g for the SGCS). The model was further employed to analyze the rate-limiting steps in the enzyme adsorption process. Although both the external-film and internal-pore mass transfer are important for enzyme adsorption on the SGCS and UGCS, the UGCS has a lower internal-pore resistance compared to the SGCS.

**Conclusions:**

Ultrafine grinding pretreatment can enhance the enzyme adsorption onto corn stover by altering structure and surface composition. The film–pore–surface diffusion model successfully captures features on enzyme adsorption on ultrafine grinding pretreated corn stover. These findings identify wherein the probable rate-limiting factors for the enzyme adsorption reside and could, therefore, provide a basis for enhanced cellulose hydrolysis processes.

## Background

Lignocellulosic biomass, such as crop residues, is the only renewable and sustainable resource that can be stored and transported. The annual yield of crop residues is abundant according to the FAO Statistics (2013), which indicated that approximately 12 % of the world’s land area is used for crop production [1]. Among the main crop residues, corn stover is one of the most favorable bioethanol feedstocks because of its wide geographic distribution and high cellulose content. The bioethanol conversion of corn stover has attracted the interest of scientists around the world [2, 3].

For the conversion of lignocellulosic biomass to bioethanol, the key bottleneck is the initial conversion of biomass to sugars. It is well known that lignocellulosic biomass, in its native form, is recalcitrant to hydrolysis with cellulase enzyme systems in the biochemical conversion process. To overcome biomass recalcitrance and improve cellulose accessibility, many chemical pretreatment methods (acid [4], alkali [5], ammonia fiber explosion [2] and so on [6]) were employed. However, these chemical pretreatment methods generate highly toxic effluents and cause negative impacts on the environment. Mechanical comminution is an environmentally friendly pretreatment that can alter the degree of polymerization, crystallinity degree, porosity and specific surface area of lignocellulosic biomass and, thus, enhance cellulose hydrolysis [7]. Most previous studies on the mechanical comminution pretreatment of lignocellulosic biomass were usually carried out by chipping (10–30 mm), grinding and milling (0.2–2 mm) [7–9].

Recently, ultrafine grinding (approximately 25 μm) technology, which can achieve a small particle size, large specific surface area, and high chemical activity [10], was also sporadically explored in the field of lignocellulose pretreatment. For example, Silva et al. investigated the effects of grinding processes on the enzymatic degradation of wheat straw [11]. The results showed that the ultrafine grinding pretreatment significantly enhanced enzymatic hydrolysis yield up to 10-fold as compared with coarsely grinding. Although some properties, such as the particle size and cellulose crystallinity, had been characterized to explain the hydrolysis mechanism after the ultrafine grinding pretreatment, some intrinsic properties, such as the adsorption kinetics, should be further investigated.

**Fig. 1:**
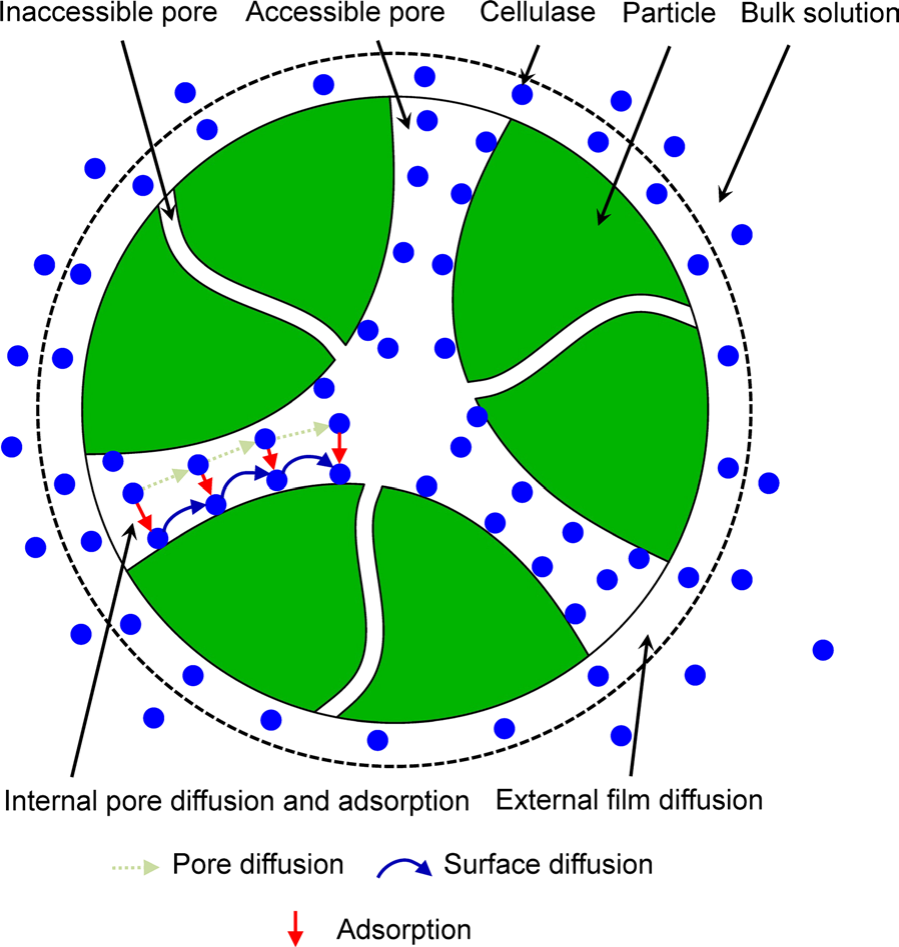
The schematic illustration of mass transfer for enzyme adsorption on a porous substrate. The adsorption of cellulase onto lignocellulosic biomass includes three consecutive steps: **a** the external diffusion of cellulase from bulk solution across the liquid film surrounding the solid biomass particles, **b** internal diffusion of cellulase through the biomass particles by pore volume diffusion and surface diffusion, and **c** the adsorption of cellulose molecules onto the biomass particles at the active sites

Cellulase adsorption onto the substrate via the binding domain is a prerequisite step for the enzymatic hydrolysis process and directly affects the enzymatic hydrolysis yield of lignocellulosic biomass [12, 13]. Thus, an adequate description of the adsorption step is indispensable for understanding and optimizing hydrolysis reaction, especially for that after the ultrafine grinding pretreatment. It is well known that ultrafine grinding increases the available specific surface area/pore volume [14] and, thus, improves the exposure level of the cellulose-binding domain, which is closely related to the cellulase adsorption kinetics. However, the cellulase adsorption kinetics of lignocellulosic biomass after the ultrafine grinding pretreatment has never been reported until now. Previous experimental and modeling studies on the cellulase adsorption of lignocellulosic biomass mainly focused on those pretreated by chemical methods, such as acid [15], hydrothermal [13], organosolv [13], and SO_2_-catalyzed steam explosion [16]. These studies commonly characterized cellulase adsorption by the Langmuir isotherm model, which describes the relationship between the amount of enzyme protein binding with substrate and the amount of enzyme protein free in solution after attaining equilibrium adsorption [17]. The Langmuir isotherm model can evaluate the maximum adsorption capacity of the substrate under different enzyme loadings, but it is not capable of expressing the adsorption kinetics of cellulase along with the adsorption time. The adsorption kinetics can be used to better understand the rate-controlling step of the mass transfer involved in the adsorption process. From a mechanistic viewpoint, the adsorption of cellulase onto lignocellulosic biomass can include three consecutive steps: the external diffusion of cellulase from bulk solution across the liquid film surrounding the solid biomass particles, internal diffusion of cellulase through the biomass particles by pore volume diffusion and surface diffusion, and the adsorption of cellulose molecules onto the biomass particles at the active sites (Fig. 1).

This study first investigated the enzyme adsorption kinetics of ultrafine grinding pretreated corn stover. Then, a film**–**pore**–**surface diffusion model was developed to explain the enzyme adsorption kinetics. The rate-limiting steps in the adsorption process were further investigated. To our knowledge, this is the first work in the literature to reveal the enzyme adsorption behavior of corn stover pretreated by ultrafine grinding, thus identifying wherein the probable rate-limiting factors for the enzyme adsorption reside and could, therefore, provide a basis for enhanced cellulose hydrolysis processes.

## Results and discussion

### Carbohydrates and lignin content

The cellulose, hemicellulose, and lignin content of the sieve-based grinding corn stover (SGCS) and ultrafine grinding corn stover (UGCS) is listed in Table 1. The amounts are similar to the previously reported values. For example, Li et al. observed 34.9 % glucan, 21.7 % xylan, and 20.5 % lignin in corn stover [18]. Saha et al. observed 37.0 % cellulose, 28.9 % hemicellulose, and 21.2 % lignin in corn stover [19]. It was also shown that there are no significant differences in the carbohydrates and lignin content of both substrates. Previous studies have noted that the chemical components have important adsorption interactions with enzyme molecules, although the enzyme adsorption of lignin is considered a nonproductive one [15]. Even if the two substrates present the similar contents, the grinding may affect the surface composition and, thus, change the adsorption capacity/affinity of the enzyme for the substrate. This is further corroborated by surface composition measurement for the two substrates. The surface areas of cellulose and lignin, which are two dominant components in the cellulase adsorption [20, 21], were measured by determining the maximum adsorption capacity of the dyes Congo Red [22] and Azure B [23] on the substrates, respectively (Table 1). The cellulose surface area of the UGCS (290.76 m^2^/g) was almost twofold higher than that of SGCS (168.69 m^2^/g). Compared with the lignin surface area of the SGCS (91.46 m^2^/g), that of the UGCS (106.7 m^2^/g) also moderately increased. These results indicated that the substrate pretreated by ultrafine grinding can induce more exposure of the surface composition (especially for cellulose), which will be favorable to enzyme adsorption.

**Table 1:** Chemical content and structural properties of SGCS and UGCS

Parameters	SGCS	UGCS
Cellulose (Mean ± SD, % dry matter)	33.44 ± 0.43	33.38 ± 0.20
Hemicellulose (Mean ± SD, % dry matter)	17.58 ± 0.19	17.47 ± 0.01
Lignin (Mean ± SD, % dry matter)	25.21 ± 0.23	24.35 ± 0.55
Particle size (Mean ± SD, *d*_50_, μm)	218.50 ± 2.12	17.45 ± 0.21
Span (Mean ± SD, (*d*_90_–*d*_10_)/*d*_50_)	2.93 ± 0.07	2.72 ± 0.01
Specific surface area (m^2^/g)	1.71	2.63
Pore volume (*V*_p_, cm^3^/g)	0.009	0.024
Accessible pore volume (*V*_pa_, cm^3^/g)	0.008	0.023
Cellulose surface area (m^2^/g)	168.69	290.76
Lignin surface area (m^2^/g)	91.46	106.70

### Particle size distribution and morphology

**Fig. 2:**
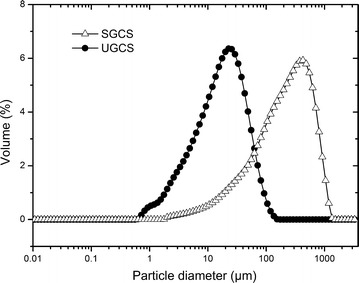
Particle size distribution of the UGCS and SGCS. These data were determined by a laser diffraction particle size analyzer

Figure 2 shows the particle size distributions of both the SGCS and UGCS. The particle size distribution was characterized by the median diameter (*d*_50_) and the span defined by (*d*_90_–*d*_10_)/*d*_50_, where *d*_10_, *d*_50_ and *d*_90_ represent the 10th, 50th and 90th percentiles of the total volume, respectively [11]. The median sizes (*d*_50_) of the UGCS and SGCS were 17.45 μm and 218.50 μm, respectively. The spans of the UGCS and SGCS were 2.72 and 2.93, respectively. The smaller span value indicated a more uniform size distribution. Severe vibration ball milling under the ultrafine grinding condition destroyed the fiber structure and, thus, achieved significant particle size reduction and unified particle size distribution. The ultrafine grinding of crop residues was also reported by several studies. For example, Silva et al. investigated the median particle sizes and particle size distribution spans of wheat straw under the operating conditions of ball milling and jet milling [11]. Ball milling reduced the particle size from 270 to 16 μm over a 0–240 h period. The span first increased to more than 5 during the first 120 h and then decreased to 2.5 at the end of the 120 h. Jet milling reduced the median particle size of wheat straw from 107 to 22 μm and was much more rapid (85 min) than ball milling. A previous study by our team also explored the ultrafine grinding of wheat straw by 8 h of vibration ball milling and reported ultrafine wheat straw powder with a median size of 17.0 μm and a span of 4.0 [24]. Compared with previous studies, our study produced ultrafine powder of corn stover in a shorter time (30 min), which indicated less energy consumption.

### Specific surface area (SSA) and pore volume (PV) distribution

**Fig. 3:**
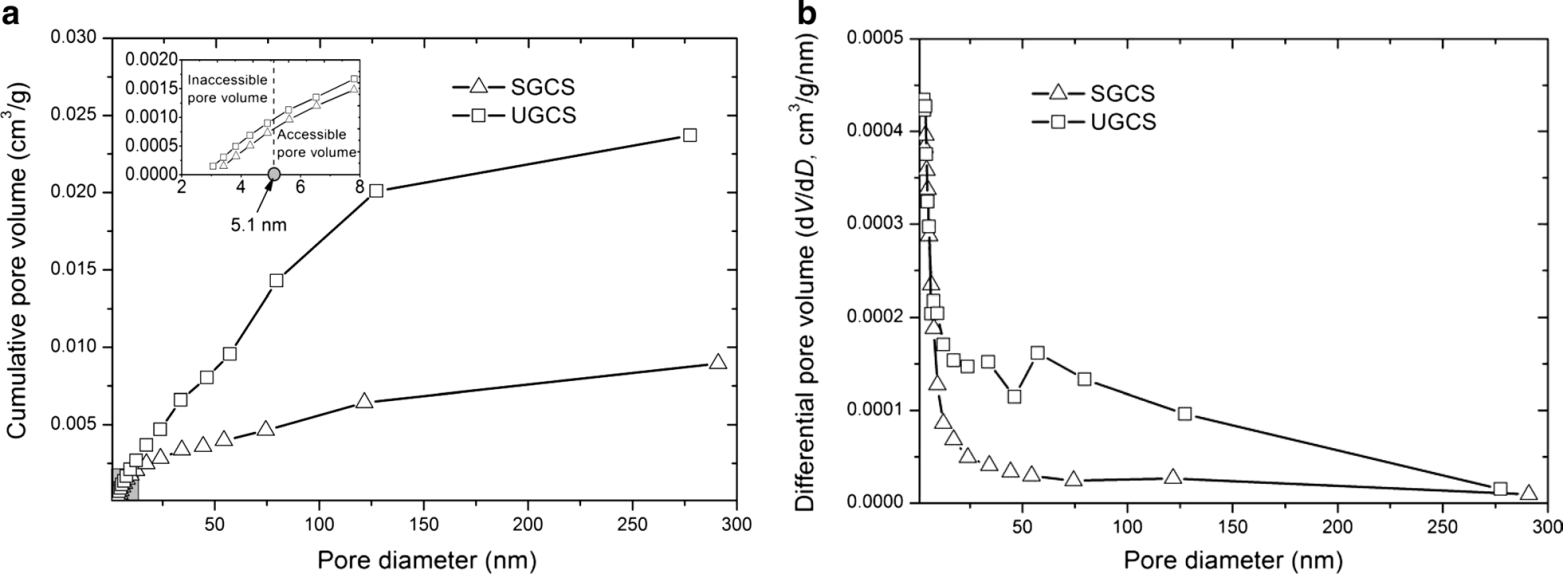
Pore volume distribution of the UGCS and SGCS as a function of pore diameter. These data were determined by liquid nitrogen adsorption experiments. **a** Cumulative pore volume versus pore diameter; **b** Differential pore volume (d*V*/d*D*) versus pore diameter

The SSA and PV distribution of the SGCS and UGCS is listed in Table 1 and Fig. 3. The SSA of the UGCS was approximately 1.5-fold higher than that of the SGCS (Table 1). Although the values between the SSA and the surface composition areas were uncomparable due to different measured methods [22], their similar increased trends for the UGCS indicated that the ultrafine grinding pretreatment significantly affects substrate structure and surface composition. The PV of the UGCS was approximately threefold higher than that of the SGCS (Fig. 3a). The UGCS had a wider pore volume distribution (2–300 nm) than the SGCS (2–50 nm) based on differential curves of the pore volume distribution (Fig. 3b), which indicated that mesopores and macropores existed in the UGCS. The SSA and PV properties are important parameters for the conversion of lignocellulosic biomass to biofuels and are often useful to ascertain whether the comminution pretreatment technology is useful or not [25]. Commonly, the comminution pretreatment can enhance the SSA of lignocellulosic biomass. This is because drastic milling to the straw can destroy the structure of the lignocellulose, disorganizing the tightly ordered fibers and exposing more enzyme bonding sites [26, 27]. Piccolo et al. found that the SSA increased by more than 60 % after ball milling compared to untreated wheat straw samples [28]. Furthermore, the SSA is highly sensitive to the particle size of lignocellulosic biomass. Zhang et al. reported a linear correlation of the SSA with particle size for pan-milling cellulose powder [29]. The SSA is not only related to the particle size, but is also strongly related to the PV of the lignocellulosic biomass. The surface area of the substrate can be divided into an interior surface area, reflected by the biomass porosity, and an exterior surface area, largely determined by the particle size. Compared with the sieve-based grinding pretreatment, the ultrafine grinding pretreatment can produce more significant changes in the internal-pore structure, and these changes are mainly responsible for the enzymatic adsorption and hydrolysis of biomass [30, 31]. The size of a cellulase is approximately 5.1 nm [32] and, hence, only those pores larger than 5.1 nm are accessible to enzyme. The pore accessible to enzyme is correlated with the enzyme diffusion resistance and adsorption rate [32, 33]. Compared with the SGCS, the UGCS has a higher volume fraction of pores larger than 5.1 nm in diameter (Fig. 3a).

### Equilibrium adsorption

**Fig. 4:**
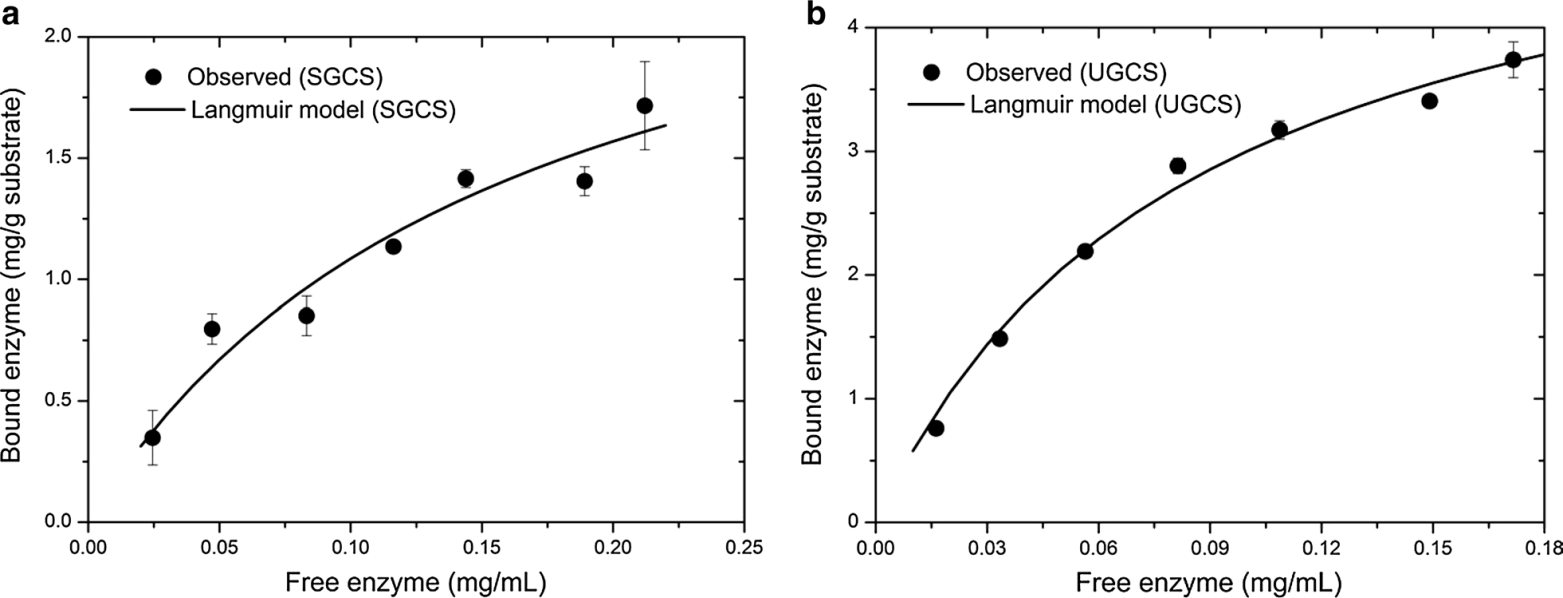
Equilibrium adsorption of cellulose to **a** SGCS and **b** UGCS. The equilibrium adsorption experiments were performed with different loadings of the cellulase (1.5–10.5 mg/g substrate for celluclast 1.5 L). The cellulase adsorption data were fitted by Langmuir equilibrium isotherm. *Error bars* represent the standard deviation of the measurements for the bound enzyme

The Langmuir isotherm model agreed well with the equilibrium adsorption data of both the SGCS and UGCS (Fig. 4) based on their statistical parameters (*R*^2^ ≥ 0.90, *RMSE* ≤ 0.20 mg/g). The Langmuir parameters, including the maximum adsorption capacity (*q*_m_), affinity constant (*K*_a_) and bonding strength (*S* = *q*_m_ × *K*_a_), are listed in Table 2. A number of previous studies carried out the cellulase equilibrium adsorption of lignocellulosic biomass and also observed robust adaptability of the Langmuir model [34]. For example, Machado et al. investigated the adsorption characteristics of cellulase on Avicel, pretreated sugarcane bagasse, and lignin [13]. Langmuir model isotherms were chosen to compare the kinetic properties of these various enzyme-substrate systems. Qi et al. explored cellulase adsorption of two different pretreated wheat straws and proposed a good fit to the cellulase adsorption data by the Langmuir adsorption isotherm [35]. It is difficult to directly compare the Langmuir parameters of this study to those of previous studies for different combinations for enzyme, substrate, and temperature. Zhang and Lynd collected Langmuir parameters for the cellulase adsorption of lignocellulosic biomass and observed wide variations [36]. However, the Langmuir parameters of both the SGCS and UGCS in this study can be directly compared because of the same experimental conditions. The results showed that the *q*_m_ (5.61 mg/g) and *K*_a_ (11.5 mL/mg) values obtained for the UGCS were much higher than those (*q*_m_ = 2.83 mg/g, *K*_a_ = 6.22 mL/mg) for the SGCS. These results indicated that the substrate pretreated by ultrafine grinding has a stronger adsorption capability of enzyme molecules. The reason for the high cellulase adsorption amount of UGCS may be because the ultrafine grinding achieved significant changes in the intact cellulose–hemicellulose–lignin network. More generated pores, demonstrated by a high SSA and PV distribution, increased the diffusion of enzyme molecules into the substrate. More importantly, more exposed binding sites of the substrate, demonstrated by a high cellulose and lignin surface area, improved the substrate accessibility to cellulase.

**Table 2:** Langmuir adsorption isotherm parameters of SGCS and UGCS

Parameters	SGCS	UGCS
Maximum solid-phase bound capacity (*q*_m_, mg protein/g substrate)	2.83	5.61
Affinity constant (*K*_a_, mL/mg protein)	6.22	11.5
Bonding strength (*S*, mL/g substrate)	17.60	64.52
*R* ^2^	0.94	0.99
*RMSE* (mg protein/g substrate)	0.12	0.11

### Film**–**pore**–**surface diffusion model for the enzymatic adsorption kinetics

**Fig. 5:**
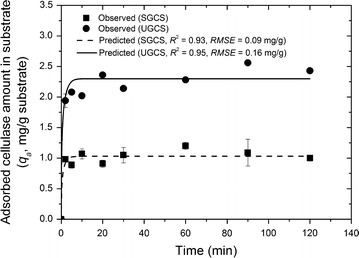
Comparison of observed and predicted cellulase adsorption kinetics for SGCS and UGCS. The cellulase adsorption kinetic experiments were performed for 2, 5, 10, 20, 30, 60, 90, and 120 min with an enzyme loading of 6 mg/g substrate. The predicted values were obtained by current film**–**pore**–**surface diffusion model. *Error bars* represent the standard deviation of the measurements for the absorbed cellulase amount in substrate

The cellulase adsorption kinetic profiles of the SGCS and UGCS are shown in Fig. 5. Compared with the kinetic data of the SGCS, the adsorption amount of the UGCS at any time was much higher. This may be explained by the changes induced by the ultrafine grinding pretreatment, which yielded high SSA, PV and surface composition areas. On the one hand, high SSA, PV and surface composition values produced a large exposure area of the substrate and, thus, the binding sites of the substrate to the cellulase are also accordingly increased to achieve high enzymatic adsorption capacity. On the other hand, large pore openings produce less restriction and provide efficient adsorption of the enzyme molecules [37]. Wang et al. [11] investigated the cellulase adsorption and cellulose accessibility to the cellulase of the set of pretreated substrates with different pore volume distributions [31]. The authors found that increasing the pore volume in the substrates increases the cellulose accessibility to cellulase, which correlated well with the amounts of adsorbed cellulase.

The film**–**pore**–**surface diffusion models were developed to simultaneously predict the cellulase adsorption kinetics of both the SGCS and UGCS (Fig. 5 and Table 3). The model prediction agreed reasonably well with the observed kinetic data based on high *R*^2^ and low *RMSE* values (*R*^2^ = 0.95 and *RMSE* = 0.16 mg/g for the UGCS, *R*^2^ = 0.93 and *RMSE* = 0.09 mg/g for the SGCS). Internal diffusion is an important mass transport process during the enzyme adsorption of corn stover particles and includes pore and surface diffusion. The fitted pore diffusion coefficients (*D*_p_) were found to be 9.45 × 10^−7^ cm^2^/min for the SGCS and 6.04 × 10^−6^ cm^2^/min for the UGCS. The magnitude of *D*_p_ is affected by pore structure parameters, such as the pore size, porosity, and tortuosity. The ultrafine grinding pretreatment can reduce the pore diffusion resistance by changing these pore structure parameters and then enhance the pore diffusion coefficient. Compared with the surface diffusion coefficient (*D*_s_) of the SGCS, that of the UGCS was smaller by several orders of magnitude. Surface diffusion is often described by a hopping mechanism in which the migrating particles are viewed as hopping between distinct, energetically favorable adsorption sites on the surface [38]. When an adsorbed particle obtains a sufficient activation energy, it can overcome the energy barrier between adsorption sites and jump to a neighboring site. Thus, the speed of surface diffusion depends on the bond strength of the attached sorption site and the affinity of the recipient site. The equilibrium adsorption data showed that the UGCS had a higher affinity constant (*K*_a_) and bonding strength (*S*) than the SGCS. This may explain the low *D*_s_ value of the UGCS. External film diffusion is another mass transfer process that is characterized by external-film transfer coefficients (*K*_L_). The fitted *K*_L_ value of the UGCS was much less than that of the SGCS. The relationship between *K*_L_ and the particle size is not straightforward. Badruzzaman et al. quantified the arsenate adsorption on granular ferric hydroxide by the film-surface diffusion model and then evaluated the *K*_L_ dependence on the particle size [39]. The results showed that the obtained *K*_L_ values did not correlate with the particle radius. The magnitude of *K*_L_ is affected not only by the adsorbent particle size but also by the adsorbent-solution system hydraulics.

**Table 3:** The kinetic and statistical parameters of the film**–**pore**–**surface diffusion model fitting

Parameters	SGCS	UGCS
*K*_L_ (cm/min)	1.53	0.14
*D*_p_ (cm^2^/min)	9.45 × 10^−7^	6.04 × 10^−6^
*D*_s_ (cm^2^/min)	3.42 × 10^−5^	1.02 × 10^−8^
*R* ^2^	0.93	0.95
*RMSE* (mg/g substrate)	0.09	0.16

To measure the relative importance of external-film mass transfer to internal-pore mass transfer within the two substrates, the Biot number (*B*_i_) was used as an indicator and calculated as *B*_i_ = *K*_L_ × *R*/*D*_e_, where *K*_L_ is the external-film transfer coefficient, *R* is the particle radius, and *D*_e_ is the effective diffusion coefficient in the internal pore. Traegner and Suidan suggested that the external-film mass transfer is the rate-controlling step for *B*_i_ < 1, both the external-film and internal-pore mass transfer are the rate-controlling steps for 1 ≤ *B*_i_ ≤ 100, and the internal-pore mass transfer is the rate-controlling step for *B*_i_ > 100 [40]. The calculated *B*_i_ values for the SGCS and UGCS were 92.81 and 39.57, respectively. Hence, these *B*_i_ values indicated that both the external-film and internal-pore mass transfer were important for cellulase adsorption on the SGCS and UGCS. However, the smaller *B*_i_ value of the UGCS indicated a lower internal-pore resistance within the UGCS. The finding showed that the ultrafine grinding pretreatment significantly decreases the particle size and improves the pore diffusion properties, such as the pore size, porosity, pore volume and pore openings, resulting in less internal-pore resistance within the UGCS.

## Conclusions

The ultrafine grinding pretreatment was executed on corn stover. The results showed that the ultrafine grinding pretreatment can significantly decrease the particle size (from 218.5 μm of SGCS to 17.45 μm of UGCS) and increase the specific surface area (SSA), pore volume (PV) and surface composition (SSA: from 1.71 m^2^/g of SGCS to 2.63 m^2^/g of UGCS, PV: from 0.009 cm^3^/g of SGCS to 0.024 m^3^/g of UGCS, cellulose surface area: from 168.69 m^2^/g of SGCS to 290.76 m^2^/g of UGCS, lignin surface area: from 91.46 m^2^/g of SGCS to 106.70 m^2^/g of UGCS). The structure and surface composition changes induced by ultrafine grinding increase the enzyme adsorption capacity from 2.83 mg/g substrate of SGCS to 5.61 mg/g substrate of UGCS. A film**–**pore**–**surface diffusion model was developed to simultaneously predict the enzyme adsorption kinetics of both the SGCS and UGCS. The model provided satisfactory predictions based on high *R*^2^ and low *RMSE* values (*R*^2^ = 0.95 and *RMSE* = 0.16 mg/g for the UGCS, *R*^2^ = 0.93 and *RMSE* = 0.09 mg/g for the SGCS). The model was further employed to analyze the rate-limiting steps in the enzyme adsorption process. Although both external-film and internal-pore mass transfer are important for the enzyme adsorption on the SGCS and UGCS, the UGCS has a lower internal-pore resistance compared to the SGCS because the ultrafine grinding pretreatment significantly decreased the particle size and improved the pore diffusion properties such as the pore size, porosity, pore volume and pore openings. These findings identify wherein the probable rate-limiting factors for the enzyme adsorption reside and could, therefore, provide a basis for enhanced cellulose hydrolysis processes.

## Methods

### Samples and enzyme preparation

Corn stover was collected in 2013 from the Shangzhuang agronomy farm of the China Agricultural University, located in Beijing, China. The corn stover was air dried and milled to coarse particle size (approximately 1–2 cm). Then, it was dried in a forced-air oven at 45 °C for 48 h and milled to a size less than 1 mm in an RT-34 hammer mill (Rong Tsong Precision Technology Co., Taiwan). The milled material was sieved by a JH-300A sieve shaker fitted with a 40-mesh screen to obtain the SGCS samples (Jiahe Machinery Co., Henan province, China). Then, 400 g of powder was further milled using a CJM-SY-B ultrafine vibration grind mill to obtain the UGCS samples (Taiji Ring Nano Products Co., Hebei, China). The corn stover powder was mixed with ZrO_2_ balls (6–10 mm diameter) in a 1:2 volume ratio for 0.5 h, and the instrument temperature was controlled below 30 °C. All powders obtained were sealed in PVC plastic bags at room temperature before use in all experiments. The celluclast 1.5 L (cellulase) was purchased from Sigma-Aldrich (St. Louis, MO, USA), and the protein content is 36.7 mg/mL.

### Analysis of the surface areas of cellulose and lignin

The surface areas of cellulose and lignin were measured according to the literature [21]. The surface areas of cellulose and lignin on the SGCS and UGCS were analyzed by determining the monolayer adsorption maximum of Congo Red (Direct Red 28) [22] and Azure B [23], respectively. Of each adsorption, 100 mg dry material was weighed in 25 mL conical flask; 10 mL of the dye (Congo Red in 30 mM phosphate buffer at pH 6 and Azure B in 50 mM Na-phosphate buffer at pH 7) was added to the conical flasks and incubated for 24 h on a shaker at 200 rpm. Congo Red adsorption was performed at 60 °C and Azure B adsorption at 25 °C. After incubation, the liquid fraction was separated by centrifugation and the supernatant was filtered through a 0.45 μm PTFE filter. The residual dye concentration and reference solutions were determined spectrophotometrically (Congo Red at 498 nm and Azure B at 647 nm) and the amounts of adsorbed dye were calculated. The adsorption experiments were performed in duplicate using Congo Red concentrations of 4, 2, 1, 0.25, 0.05 and 0 g/L and Azure B concentrations of 2, 1, 0.5, 0.25, 0.1 and 0 g/L. The parameters of the adsorption isotherm were fitted to the Langmuir isotherm in MATLAB (Mathworks, Natick, MA, USA). Then, the cellulose surface area was calculated per dry material from the adsorption maximum with 1 g of the adsorbed dye representing 1055 m^2^ surface [22]. And the surface area of the lignin was obtained from the maximum adsorption capacity and the area (1.297 m^2^/mg) covered by Azure B [23].

### Particle size determination

The particle size distribution was measured using an LS230 laser diffraction particle size analyzer (Beckman Coulter Inc., Miami, FL, USA). The particle measurement range is from 0.375 μm to 2000 μm. Before measurement, the samples were dispersed with distilled water to form a uniform liquid suspension and then were poured into the measurement instrument with ultrasound. An LS v3.29 system based on the Fraunhofer mode was used to measure the particle size.

### Specific surface area, pore size and pore volume distribution determination

The specific surface area, pore size and pore volume distribution of the SGCS and UGCS were measured with the Autosorb-iQ porosity analyzer (Quantachrome Instruments, FL, USA). The samples were degassed at 80 °C for 7 h and then cooled in the presence of nitrogen gas under −195 °C, allowing the nitrogen gas to condense on the surfaces and within the pores. The specific surface area was calculated using the Brunauer–Emmett–Teller (BET) model [41], which relates the gas pressures to the volume of gas adsorbed. The pore volume distribution with respect to the pore size was estimated using the Barrett–Joyner–Halenda (BJH) model [42].

### Enzyme adsorption kinetic experiments

The enzyme adsorption kinetic experiments were conducted for the SGCS and UGCS with an enzyme loading of 6 mg protein/g substrate, which was among the usual enzyme hydrolysis loading capacity. The lignocellulose substrate-binding studies were performed in centrifuge tubes (10 mL) with a sodium citrate buffer (0.05 M, pH 4.8) using a 1 % (*w/v*) substrate concentration and incubated for 2, 5, 10, 20, 30, 60, 90, and 120 min in a shaking water bath at 4 °C to avoid hydrolysis. Every experiment was run two times, and substrate blanks without enzyme and enzyme blanks without substrate were also analyzed. After incubation, all samples were centrifuged for 3 min in a refrigerated centrifuge at 6000 rpm. The supernatant was filtered and used to determine the free enzyme by measuring the protein concentration in the supernatant using the Bradford assay by Coomassie brilliant blue dye [43]. The bound enzyme was calculated by subtracting the free enzyme concentration from the initial enzyme concentration loaded.

### Equilibrium enzyme adsorption experiments

Different loadings of the enzyme (1.5–10.5 mg/g substrate for celluclast 1.5 L) were performed and incubated for 2 h under the same condition mentioned above. The bound enzyme concentration calculated was correlated with the free enzyme concentration using the following Langmuir equilibrium isotherm:

1 $$q_{\text{b}} = \frac{{q_{\text{m}} K_{\text{a}} C_{\text{f}} }}{{ 1+ K_{\text{a}} C_{\text{f}} }}$$

where *q*_b_ is the equilibrium amount of solid-phase bound enzyme (mg protein/g substrate), *q*_m_ is the maximum solid-phase bound capacity (mg protein/g substrate), *K*_a_ is the affinity constant (mL/mg protein), and *C*_f_ is the equilibrium concentration of free enzyme in solution (mg protein/mL).

The Langmuir adsorption constants (*K*_a_ and *q*_m_) of the SGCS and UGCS were obtained by nonlinear regression using MATLAB (Mathworks, Natick, MA, USA). The binding strength (*S* in mL/g substrate), another constant from the Langmuir adsorption isotherm, could be used to estimate the stability of the enzyme bound with substrates. The binding strength can be calculated by *S* = *q*_m_ × *K*_a_.

### Film**–**pore**–**surface diffusion adsorption model

The adsorption of cellulase onto lignocellulosic biomass involves three consecutive steps: external diffusion of the cellulase from the bulk solution across the liquid film surrounding the solid biomass particles, internal diffusion of the cellulase through the biomass particles by pore volume diffusion and surface diffusion, and the adsorption of cellulose molecules onto the biomass particles at the active sites (Fig. 1). The film**–**pore**–**surface diffusion adsorption model was proposed based on the following assumptions: (a) the adsorbent particles are spherical; (b) the adsorption rate at an active site is instantaneous; and (c) the solute adsorbed amount on the adsorbent can be represented by the Langmuir isotherm equation.

The rate of mass transfer in the external film surrounding the solid particle is assumed to be directly proportional to the concentration difference in the film. Therefore, the external-film mass transfer is given by

2 $$V_{\text{L}} \frac{{dC_{\text{L}} }}{dt} = - K_{\text{L}} A(C_{\text{L}} - C_{\text{P,r}} \left| {_{\text{r = R}} } \right.)$$

where *t* is the adsorption time, *V*_L_ is the total volume of the liquid phase, *C*_L_ is the concentration of enzyme in the liquid phase, *C*_P,r_|_r=R_ is the enzyme concentration at the particle surface, *K*_L_ represents the external-film mass transfer coefficient, and *A* represents the outer surface area of all the particles, estimated as:

3 $$A = \frac{3m}{{\rho_{\text{a}} R}}$$

where *m* is the mass of all the particles, *R* is the radius of the particle, and *ρ*_a_ is the apparent density of the particle, estimated as:

4 $$\rho_{\text{a}} = \frac{{\rho_{\text{s}} }}{{1 + V_{\text{p}} \rho_{\text{s}} }}$$

where *V*_p_ is the pore volume per mass of the particle and *ρ*_s_ is the solid density, estimated as follows:

5 $$\rho_{\text{s}} = \frac{1}{{M_{\text{c}} /\rho_{\text{c}} + M_{\text{h}} /\rho_{\text{h}} + M_{\text{l}} /\rho_{\text{l}} + M_{\text{o}} /\rho_{\text{o}} }}$$

where *M*_c_, *M*_h_, *M*_l_, and *M*_o_ and *ρ*_c_, *ρ*_h_, *ρ*_l_, and *ρ*_o_ are the mass percentages on a dry basis and the densities of cellulose, hemicellulose, lignin and other compositions in solid particles, respectively.

Based on the mass balance equation for the adsorption of enzyme with internal-pore diffusion in a spherical particle, the following equation can be obtained:

6 $$\begin{aligned}\varphi \varepsilon \frac{{\partial C_{\text{P,r}} }}{\partial {t}} + \rho_{\text{a}} \frac{{\partial {q}_{\text{r}} }}{\partial {t}} &= \frac{1}{{r^{2} }}\frac{\partial }{\partial {r}}\left[ {r^{2} {D}_{\text{p}} \frac{{\partial {C}_{\text{P,r}} }}{\partial {r}}} \right] \\ & \quad + \frac{1}{{r^{2} }}\frac{\partial }{\partial {r}}\left[ {r^{2} \rho_{\text{a}} D_{\text{s}} \frac{{\partial {q}_{\text{r}} }}{\partial {r}}} \right] \end{aligned}$$

where *C*_P,r_ is the enzyme concentration in the particle pores at position *r*, $\varphi$ is the ratio of the accessible pore volume to the enzyme (*V*_pa_) to the total pore volume (*V*_p_), *r* is the radial position in the particle, *q*_r_ is the solid-phase enzyme adsorption amount at position *r*, *D*_p_ is the pore diffusion coefficient of the enzyme, *D*_s_ is the surface diffusion coefficient of the enzyme, and *ε* is the porosity of the solid particle, estimated as:

7 $$\varepsilon = \frac{{V_{\text{p}} }}{{V_{\text{p}} + 1/\rho_{\text{s}} }}$$

As the adsorption step occurs much more rapidly than the mass transfer step in physical adsorption, the pore solution concentration and the solid-phase adsorbed amount can be expressed by the Langmuir isotherm equation:

8 $$q_{\text{r}} = f(C_{\text{P,r}} ) = \frac{{q_{\text{m}} K{}_{\text{a}}C_{\text{P,r}} }}{{1 + K_{\text{a}} C_{\text{P,r}} }}$$

Differentiating Eq. (8) yields:

9 $$dq_{\text{r}} = f^{\prime } (C_{\text{P,r}} )dC_{\text{P,r}}$$

Substituting Eq. (9) into Eq. (6) gives:

10 $$\left[ {\varphi \varepsilon + \rho_{\text{a}} f^{\prime } (C_{\text{P,r}} )} \right]\frac{{\partial C_{\text{P,r}} }}{\partial t} = \frac{1}{{r^{2} }}\frac{\partial }{\partial r}\left[ {r^{2} D_{\text{e}} \frac{{\partial C_{\text{P,r}} }}{\partial r}} \right]$$

where *D*_e_ is the effective diffusion coefficient in the internal pore, given as:

11 $$D_{\text{e}} = D_{p} + f^{\prime}(C_{\text{P,r}} )\rho_{\text{a}} D_{\text{s}}$$

Substituting $f^{'} (C_{\text{P,r}} ) = \frac{{q_{\text{m}} K_{\text{a}} }}{{(1 + K_{\text{a}} C_{\text{P,r}} )^{2} }}$ into Eq. (10) gives:

12 $$\begin{aligned} \left( {\varphi \varepsilon + \frac{{\rho_{\text{a}} q_{\text{m}} K_{\text{a}} }}{{(1 + K_{\text{a}} C_{\text{P,r}} )^{2} }}} \right)\frac{{\partial C_{\text{P,r}} }}{\partial t} &= \hfill \left( {D_{\text{p}} + \frac{{D_{\text{s}} \rho_{\text{a}} q_{\text{m}} K_{\text{a}} }}{{(1 + K_{\text{a}} C_{\text{P,r}} )^{2} }}} \right)\frac{{\partial^{2} C_{\text{P,r}} }}{{\partial r^{2} }} \\ & \quad- \frac{{2q_{\text{m}} K_{\text{a}}^{2} D_{\text{s}} \rho_{\text{a}} }}{{(1 + K_{\text{a}} C_{\text{P,r}} )^{3} }}\left( {\frac{{\partial C_{\text{P,r}} }}{\partial r}} \right)^{2} + \frac{2}{r}\left( {D_{\text{p}} + \frac{{D_{\text{s}} \rho_{\text{a}} q_{\text{m}} K_{\text{a}} }}{{(1 + K_{\text{a}} C_{\text{P,r}} )^{2} }}} \right)\frac{{\partial C_{\text{P,r}} }}{\partial r} \hfill \\ \end{aligned}$$

The average enzyme adsorption amount in the solid particles (*q*_a_) is given by

13 $$q_{\text{a}} = \frac{{\int_{0}^{R} {q_{\text{r}} \times } 4\pi r^{2} dr}}{{\frac{4}{3}\pi R^{3} }} = \frac{3}{{R^{3} }}\int_{0}^{R} {q_{\text{r}} r^{2} } dr$$

The initial and boundary conditions are listed as:

14 $$t = 0\begin{array}{*{20}c} {} & {C_{\text{L}} = C_{0} } \\ \end{array} \begin{array}{*{20}c} {} & {\begin{array}{*{20}c} {C_{\text{p,r}} \left| {_{0 \le r \le R} } \right. = 0} & {} \\ \end{array} } \\ \end{array}$$

15 $$\begin{aligned} t > 0 & {K_{\text{L}} (C_{\text{L}} - C_{\text{P,r}} \left| {_{\text{r = R}} } \right. )= D_{\text{e}} \frac{{\partial C_{\text{P,r}} }}{\partial r}\text{|}_{\text{r = R}} } \\&\quad {\frac{{\partial C_{\text{P,r}} }}{\partial r}\text{|}_{\text{r = 0}} = 0} \end{aligned}$$

The film**–**pore**–**surface diffusion model can be numerically solved by combining Eqs. (2–15). The *C*_P,r_ values can be used to calculate the average enzyme adsorption amount in the solid particles (*q*_a_) according to Eqs. (8) and (13). The predicted *q*_a_ values were compared with the observed values and were used to estimate the model parameters. The model parameters (*K*_L_, *D*_p_, and *D*_s_) were simultaneously fitted to all experimental data using a custom-written program in MATLAB (Mathworks, Natick, MA, USA). Table 4 provides a description of these symbols.

**Table 4:** The model symbols

Parameters	Description	Units	Value		Sources
			SGCS	UGCS	
*V*_L_	Total volume of the liquid phase	mL	5	5	This study
*C*_0_	Initial concentration of the enzyme in the bulk solution	mg/mL	0.133	0.133	This study
*C*_L_	Enzyme concentration in the bulk solution	mg/mL	Dep. var.	Dep. var.	–
*C*_P,r_|_r=R_	Enzyme concentration at the particle surface	mg/mL	Dep. var.	Dep. var.	–
*C*_P,r_	Enzyme concentration in the particle pores at position *r*	mg/mL	Dep. var.	Dep. var.	–
*q*_b_	Equilibrium amount of solid-phase bound enzyme	mg/g substrate	Dep. var.	Dep. var.	–
*q*_m_	Maximum solid-phase bound capacity	mg/g substrate	Indep. var.	Indep. var.	–
*K*_a_	Affinity constant	mL/mg	Indep. var.	Indep. var.	–
*C*_f_	Equilibrium concentration of free enzyme in solution	mg/mL	Indep. var.	Indep. var.	–
*t*	Adsorption time	min	Indep. var.	Indep. var.	–
*A*	Total outer surface area of all the particles	cm^2^	Dep. var.	Dep. var.	–
*R*	Radius of the particle	cm	2.18 × 10^−2^	0.18 × 10^−2^	This study
*m*	Mass of all the particles	g	0.1	0.1	This study
*r*	Radial position in the particle	cm	Indep. var.	Indep. var.	–
*ρ*_a_	Apparent density of the particle	g/cm^3^	Dep. var.	Dep. var.	–
*ρ*_s_	Solid density of the particle	g/cm^3^	Indep. var.	Indep. var.	–
*V*_pa_	Pore volume accessible to the enzyme	cm^3^/g	Indep. var.	Indep. var.	–
*V*_p_	Pore volume	cm^3^/g	Indep. var.	Indep. var.	–
*φ*	Ratio of the pore volume accessible to the enzyme to the total pore volume	–	Dep. var.	Dep. var.	–
*K*_L_	External-film mass transfer coefficient	cm/min	Indep. var.	Indep. var.	–
*D*_p_	Pore diffusion coefficient of the enzyme	cm^2^/min	Indep. var.	Indep. var.	–
*D*_s_	Surface diffusion coefficient of the enzyme	cm^2^/min	Indep. var.	Indep. var.	–
*D*_e_	Effective diffusion coefficient in the internal pore	cm^2^/min	Dep. var.	Dep. var.	–
*q*_r_	Solid-phase enzyme adsorption amount at position *r*	mg/g substrate	Dep. var.	Dep. var.	–
*q*_a_	Average enzyme adsorption amount in the solid particles	mg/g substrate	Dep. var.	Dep. var.	–
*ε*	Porosity	–	Dep. var.	Dep. var.	–
*M*_c_	Mass percentage of cellulose in the particles	%	33.44	33.38	This study
*M*_h_	Mass percentage of cellulose in the particles	%	17.58	17.47	This study
*M*_l_	Mass percentage of lignin cellulose in the particles	%	25.21	24.35	This study
*M*_o_	Mass percentage of other compositions such as ash in the particles	%	23.77	24.8	This study
*ρ*_c_	Cellulose density	g/cm^3^	1.52	1.52	[44]
*ρ*_h_	Hemicellulose density	g/cm^3^	1.56	1.56	[44]
*ρ*_l_	Lignin density	g/cm^3^	1.39	1.39	[44]
*ρ*_o_	Density of other compositions such as ash	g/cm^3^	2.50	2.50	[45]

Dependent variables are listed as Dep. var. and can be calculated from one of the equations while independent variables are listed as Indep. var

## Abbreviations

RMSE: root mean squared error
SGCS: sieve-based grinding corn stover
UGCS: ultrafine grinding corn stover
PV: pore volume
SSA: specific surface area
